# Functional prostate-specific membrane antigen is enriched in exosomes from prostate cancer cells

**DOI:** 10.3892/ijo.2014.2256

**Published:** 2014-01-10

**Authors:** TIANCHENG LIU, DESIREE E. MENDES, CLIFFORD E. BERKMAN

**Affiliations:** Department of Chemistry, Washington State University, Pullman, WA 99164, USA

**Keywords:** prostate-specific membrane antigen, exosome, biomarker, glycosylation, prostate cancer

## Abstract

Developing simple and effective approaches to detect tumor markers will be critical for early diagnosis or prognostic evaluation of prostate cancer treatment. Prostate-specific membrane antigen (PSMA) has been validated as an important tumor marker for prostate cancer progression including angiogenesis and metastasis. As a type II membrane protein, PSMA can be constitutively internalized from the cell surface into endosomes. Early endosomes can fuse with multivesicular bodies (MVB) to form and secrete exosomes (40–100 nm) into the extracellular environment. Herein, we tested whether some of the endosomal PSMA could be transferred to exosomes as an extracellular resource for PSMA. Using PSMA-positive LNCaP cells, the secreted exosomes were collected and isolated from the cultured media. The vesicular structures of exosomes were identified by electron microscopy, and exosomal marker protein CD9 and tumor susceptibility gene (TSG 101) were confirmed by western blot analysis. Our present data demonstrate that PSMA can be enriched in exosomes, exhibiting a higher content of glycosylation and partial proteolysis in comparison to cellular PSMA. An *in vitro* enzyme assay further confirmed that exosomal PSMA retains functional enzymatic activity. Therefore, our data may suggest a new role for PSMA in prostate cancer progression, and provide opportunities for developing non-invasive approaches for diagnosis or prognosis of prostate cancer.

## Introduction

Prostate cancer remains the second leading cause of cancer death for men in the US. According to the National Cancer Institute, it is estimated that there will be 238,590 new cases and 29,720 deaths from prostate cancer in 2013 (http://www.cancer.gov/cancertopics/types/prostate). The cell-surface enzyme prostate-specific membrane antigen (PSMA) is upregulated and strongly expressed on prostate cancer cells, including those that are metastatic (1). Endothelial-expression of prostate-specific membrane antigen (PSMA) in the neovasculature of a variety of non-prostatic solid malignancies has also been reported (2,3). PSMA is a type II membrane protein, consisting of a cytoplasmic domain (1–19aa), transmembrane domain (20–44aa) and extracellular domain (45–750aa), exhibits both N-acetylated α-linked acidic dipeptidase (NAALADase) and folate hydrolase (FOLH) activities, and constitutive or induced internalization (4–6). These properties have allowed PSMA to attract considerable attention as a target for antibody or small molecule inhibitor-guided delivery of imaging and therapeutic agents toward prostate cancer (7–15). Furthermore, pilot studies support the position that PSMA is an ideal biomarker for the targeted imaging and therapy of PSMA-positive prostate cancer.

Exosomes are small vesicles (40–100 nm) secreted by multiple normal tissue or pathological cells including cancers containing proteins, mRNAs, microRNAs and lipids that are from original cells (16). Through exosome-carrying messages, cells can achieve cross-talk without contacting each other (17). It was noted that there are elevated exosome levels secreted by highly advanced cancer cells enriched with tumor-markers (18). These studies may suggest that tumor-secreting exosomes may play an important role in the development and progression of cancer, serving as a potential biomarker resource to develop non-invasive and dynamic approaches for tumor diagnosis and prognostic evaluation of cancer treatment (16–19).

In the present study, we sought to determine the extent of PSMA enrichment in exosomes secreted by human prostate cancer cells (PSMA-positive LNCaP), and whether the exosomal PSMA is functionally active. Herein, our data revealed that tumor-marker PSMA is strongly enriched in exosomes secreted by PSMA-positive prostate cancer cells, and the exosomal PSMA maintains its functional enzymatic activity despite of higher glycosylation content. Therefore, tumor-related exosomal PSMA may serve as a diagnostic or prognostic biomarker for prostate cancer.

## Materials and methods

### Cell lines and reagents

The human prostate cancer cell line LNCaP was obtained from the American Type Culture Collection (Manassas, VA, USA). CWR22Rv1 cells were obtained from Professor Henry F. VanBrocklin (University of California, San Francisco, CA, USA). Mouse monoclonal anti-human EpCAM antibody was obtained from Cell Signaling Technology (Danvers, MA, USA). Mouse monoclonal anti-TSG 101 antibody (C-2) was obtained from Santa Cruz Biotechnology (Santa Cruz, CA, USA). Mouse monoclonal anti-GAPDH antibody and anti-α-tubulin antibody were obtained from Sigma-Aldrich (St. Louis, MO, USA). Mouse monoclonal anti-CD9 antibody (LT-86A) was a gift of Dr Davis at Washington State University (Pullman, WA, USA). Mouse monoclonal anti-PSMA antibody 7E11 was graciously provided by Cytogen Corporation (Princeton, NJ, USA). PNGase F was obtained from New England Biolabs (Ipswich, MA, USA). Halt Protease Inhibitor Cocktail (100X) was purchased from Thermo Fisher Scientific (Rockford, IL, USA). All other chemicals and cell-culture reagents were purchased from Fisher Scientific (Sommerville, NJ, USA) or Sigma-Aldrich.

### Cell culture

LNCaP and CWR22Rv1 were grown in T-75 flasks with normal growth media [RPMI-1640 containing 10% heat-inactivated fetal bovine serum (FBS), 100 units of penicillin and 100 *μ*g/ml streptomycin] in a humidified incubator at 37°C with 5% CO_2_. Confluent cells were detached with a 0.25% trypsin 0.53 mM EDTA solution for subculture growth.

### Exosome isolation

Twenty flasks (each cell line) of prostate cancer cells at 80% confluence (3 days), were subjected to washing twice in 5 ml serum-free RPMI-1640 media, then replaced with 10 ml serum-free RPMI-1640 media to continue growth for 48 h. A total of 100 ml of cell conditioned media were centrifuged at 300 × g for 10 min at 4°C to pellet the suspension cells. The supernatant media was further cleared by centrifugation at 16,500 × g at 4°C for 30 min to remove protein aggregates and cell debris. The collected supernatant was passed through 0.22 *μ*m filter to remove the >200 nm protein aggregates or vesicles. The filtered media was concentrated to 60 ml using a 70 kDa MWCO Centricon Plus-20 filter capsule (Millipore, Billerica, MA, USA). The concentrated media were transferred to an ultracentrifuge tube for centrifugation at 100,000 × g for 70 min at 4°C to pellet exosomes. The isolated exosomes were rinsed in 10 ml of PBS buffer, and centrifuged at 100,000 × g for 1 h at 4°C to be applied for the following experiments.

### Transmission electron microscopy (TEM)

The isolated exosomes from LNCaP cells were fixed with 50 *μ*l of 4% formaldehyde for 15 min at room temperature, and 5 *μ*l of sample was loaded onto carbon-coated copper grids and left for 20 min at room temperature. The sample was washed three times in PBS and then fixed for 5 min in 1% glutaraldehyde. After three washes, the exosome sample was stained for 10 min with saturated aqueous uranyl, and dried after removal of excess liquid. The samples were observed in a FEI Tecnai T-20 at 200 kV and images were recorded using iTEM software (Olympus, Münster, Germany).

### Exosomal protein extraction and western blot analysis

For protein extraction, the isolated exosomes were re-suspended in 50 *μ*l of lysis buffer (1% NP-40, 20 mM Tris pH 8.0, 137 mM NaCl, 10% glycerol) supplemented with 1X Halt Protease Inhibitor Cocktail, kept on ice for 15 min, then centrifuged at 10,000 × g for 15 min. The supernatant was collected, and stored at −80°C. The whole-cell protein extraction was also performed as controls, according to our previous protocol (20–22). Protein concentrations were determined using Non-Interfering Protein Assay (G-Biosciences, St. Louis, MO, USA). Western blot analysis was performed as described previously with only minor modifications (22,23). In brief, cellular proteins (30 *μ*g) and exosomal proteins (5 *μ*g) were loaded and separated on a NuPAGE™ 4–12% Bis-Tris Gel (Invitrogen, Carlsbad, CA, USA) by electrophoresis for 60 min at a constant 200 V under reducing conditions, and then transferred to a 0.45-*μ*m PVDF Immobilon-P Transfer Membrane (Millipore Corporation, Bedford, MA, USA) at 400 mA for 120 min in a transfer apparatus-Owl Bandit VEP-2 (Owl, Portsmouth, NH, USA) according to the manufacturer’s instructions. Membranes were incubated with primary antibody at corresponding dilution overnight at 4°C and then with horseradish peroxidase conjugated-second antibody for 1 h at room temperature. The immunoreactive bands were visualized using Protein Detector TMB Western Blot Kit (KPL, Gaithersburg, MD, USA) following the manufacturer’s instructions.

### Deglycosylation analysis of PSMA

According to manufacturer’s guidance, cellular PSMA and exosomal PSMA were subjected to denaturation in 1X denaturing buffer for 10 min at 100°C, cooled and spun down. The denatured proteins were mixed with PNGase F in 1X reaction buffer containing 1% NP-40 to incubate for 3 h at 37°C. Suitable amounts of deglycosylated PSMAs were analyzed by western blotting, the equal amounts of intact cellular PSMA and exosomal PSMA were loaded as controls.

### Enzymatic activity analysis

HPLC-based PSMA enzymatic activity analysis was performed in triplicate as described previously with only minor modifications (6,24). Working solutions of the substrate {N-[4-(phenylazo)-benzoyl]-glutamyl-γ-glutamic acid, (PABGγG)} were made at 10 *μ*M in Tris buffer (50 mM, pH 7.4). Working solutions of each protein sample were diluted at suitable concentrations in Tris buffer (50 mM, pH 7.4 containing 1% Triton X-100) to obtain ∼15% conversion (product/total substrate). A typical incubation mixture (final volume 250 *μ*l) was prepared by the addition of 175 *μ*l Tris buffer (50 mM, pH 7.4) and PABGγG (25 *μ*l, 10 *μ*M) in a test tube. The enzymatic reaction was initiated by the addition of 25 *μ*l of the PSMA working solution. The reaction was allowed to proceed for 15 min with constant shaking at 37°C and terminated by the addition of 25 *μ*l methanolic TFA (2.5% trifluoroacetic acid by volume in methanol) followed by vortexing. The quenched incubation mixture was quickly buffered by the addition of 25 *μ*l K_2_HPO_4_ (0.1 M), vortexed, iced for 15 min, and centrifuged (10 min at 7,000 × g). An 85 *μ*l aliquot of the resulting supernatant was subsequently quantified for the proportions of substrate and product by HPLC as previously described (25,26). Fractional enzymatic activity for each protein sample was calculated from HPLC data. The relative enzymatic activity (exosomal PSMA/cellular PSMA) was further calculated.

## Results

### Validation of exosome isolation protocol

**Figure 1.**
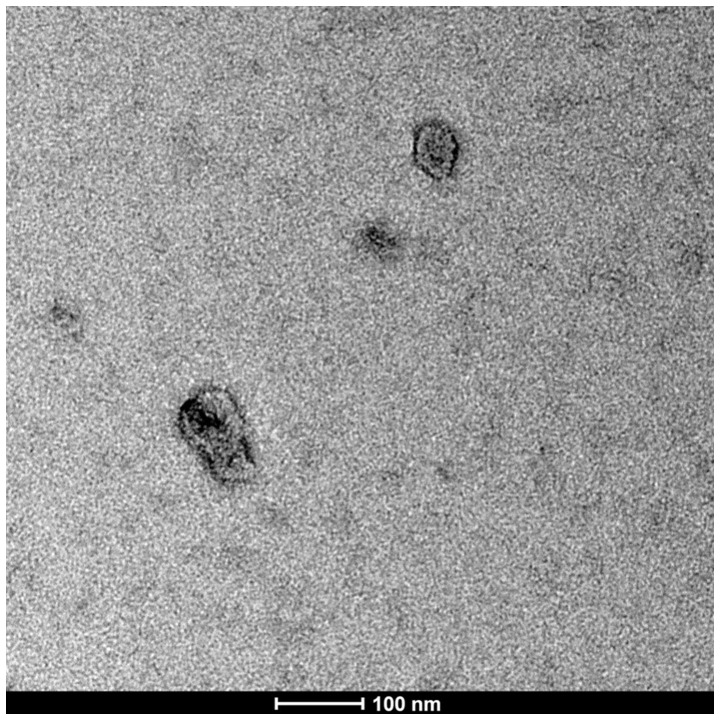
TEM analysis of exosomes from LNCaP cells. Exosomes exhibit classic morphology with round or cup-shaped membrane vesicles (40–100 nm). Distance scale, 100 nm.

TEM analysis clearly revealed that vesicle morphology was cup or round shaped and the size (<100 nm) was characteristic (27,28) for exosomes (Fig. 1). This result confirmed the efficacy of our protocol for isolating exosomes from LNCaP cells.

### Enrichment of PSMA in exosomes

**Figure 2.**
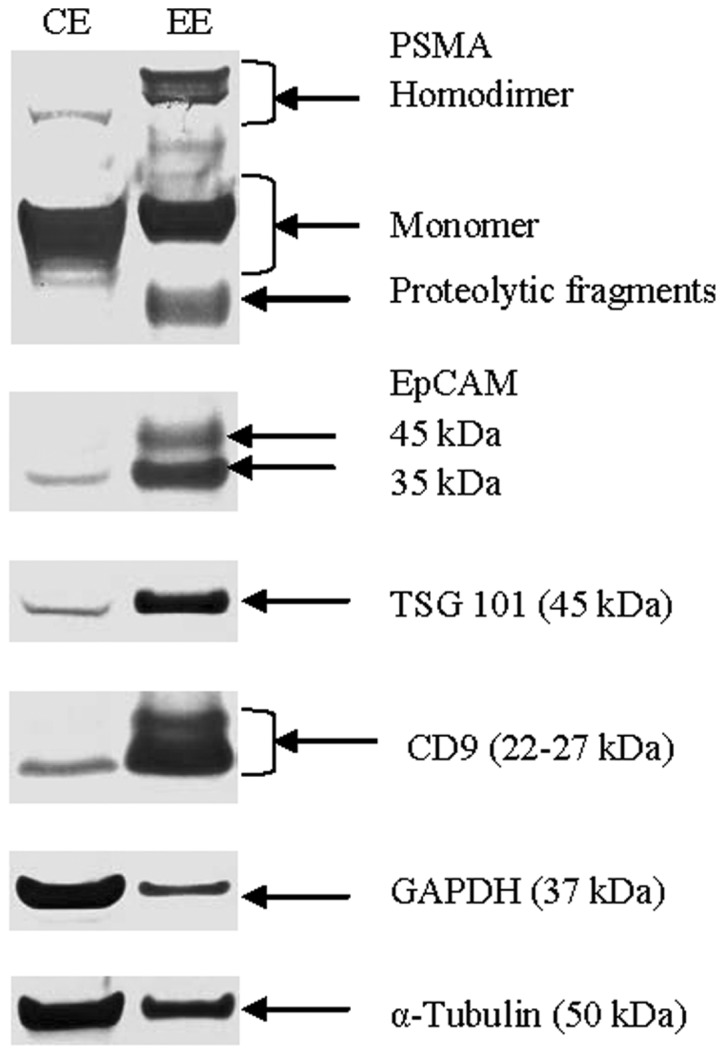
Differently enriched proteins by LNCaP cells and exosomes. The cell extract (CE) and exosome extract (EE) were analyzed by western blotting. The data clearly demonstrated that exosomes were moderately enriched with PSMA, and high levels for exosomal markers (TSG 101, CD9) and EpCAM, a low level of GAPDH and relatively stable for α-tubulin, which was detected to serve as a protein loading control.

Western blot analysis data (Fig. 2) demonstrated exceptional enrichment of exosomal markers (TSG 101, CD9) and epithelial cell adhesion molecule (EpCAM), as well as moderately enriched prostate tumor-marker PSMA in exosomes when compared to relatively stable α-tubulin levels in cells and exosomes. Interestingly, exosomal PSMA was also found to be highly enriched with an increase in molecular weight when compared to the cell extract, and also contained a small amount of proteolytic fragments (Fig. 2).

### Highly glycosylated exosomal PSMA

**Figure 3.**
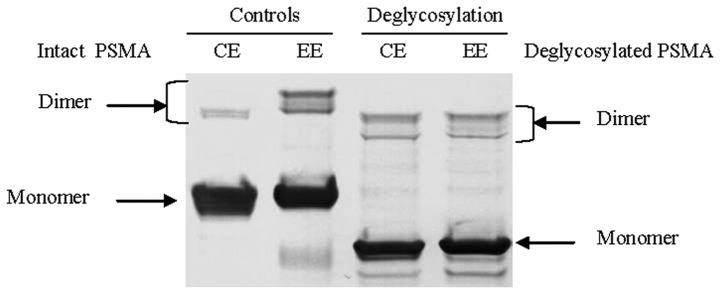
Deglycosylation analysis of cellular and exosomal PSMAs. The cell extract (CE) and exosome extract (EE) proteins were deglycosylated with PNGase F and analyzed by western blotting. Deglycosylated PSMAs exhibit the same size of molecular weight, implicating that high-content glycosylation contributes to the increased molecular weight of exosomal PSMA, compared to cellular PSMA.

To identify the source of PSMA’s perturbed molecular weight, glycosylation analysis of cellular and exosomal PSMAs were performed with PNGase F to remove all N-linked glycosylation from PSMA. After deglycosylation, cellular and exosomal PSMAs exhibited the same size of molecular weight on western blot analysis (Fig. 3).

### Retaining enzymatic activity of exosomal PSMA

**Figure 4.**
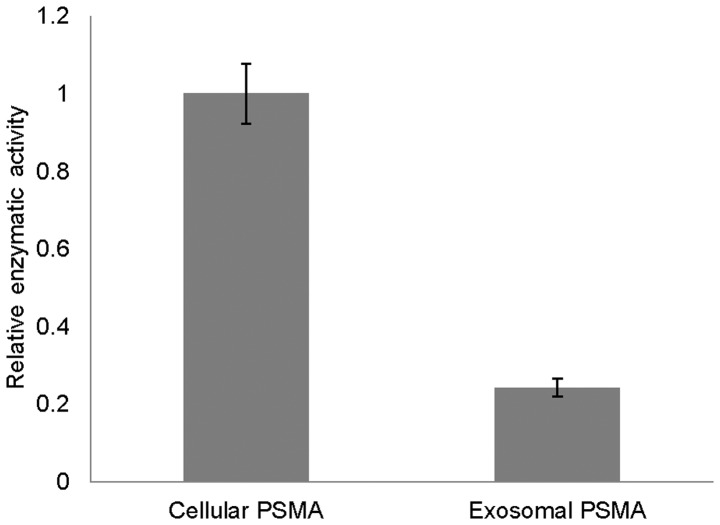
Decreased enzyme activity of exosomal PSMA. Relative enzymatic activity (exosomal PSMA: cellular PSMA at equal protein level) is about 0.24. The decreased enzyme activity is due to partial proteolysis or low pH-mediated denaturation of internalized PSMA during exosome formation.

Equal amounts of exosomal and cellular PSMAs were evaluated for their enzymatic activities using an HPLC-based, *in vitro* enzyme assay. Exosomal PSMA retains ∼24% enzyme activity of cellular PSMA (Fig. 4), attributed to partial proteolysis (Fig. 2) and lower pH within endosomes causing denaturation of internalized PSMA during exosome formation.

### Confirmation of enriched PSMA in CWR22Rv1-derived exosomes

**Figure 5.**
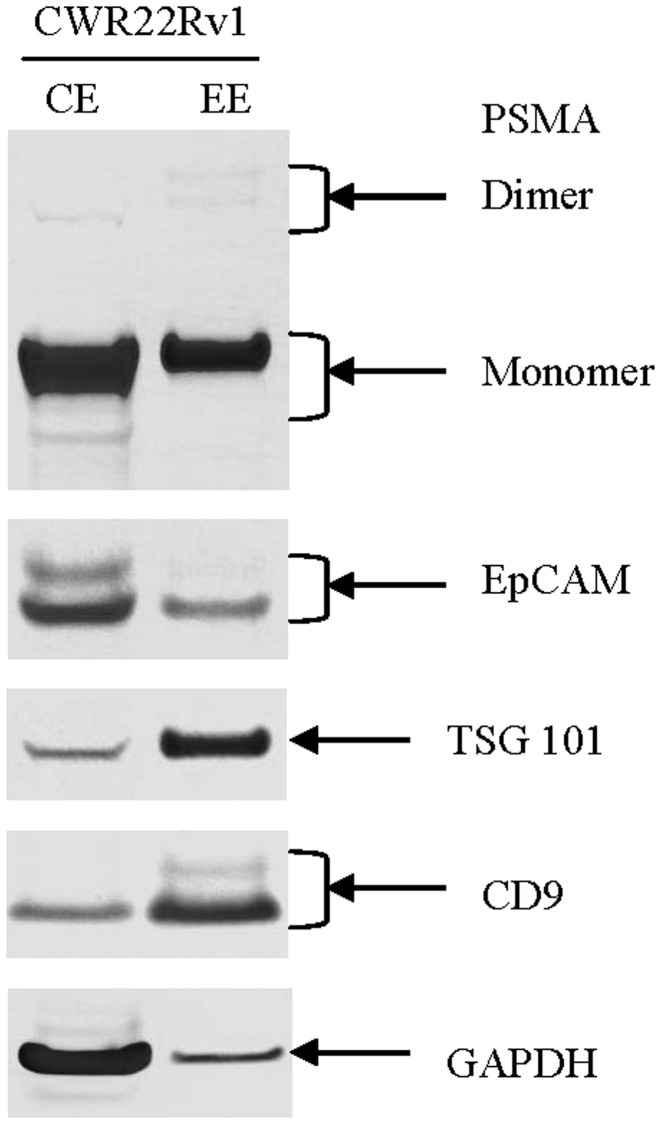
Confirmation of enriched PSMA in CWR22Rv1-derived exosomes. The cell extract (CE) and exosome extract (EE) were analyzed by western blotting. The data clearly demonstrated that CWR22Rv1-derived exosomes were moderately enriched with PSMA in the same manner as LNCaP-derived exosomes, high levels for exosomal markers (TSG 101, CD9), and low levels for EpCAM and GAPDH.

Employment of another PSMA-positive prostate cancer cell line (CWR22Rv1) through western blot analysis (Fig. 5) further validated that CWR22Rv1-derived exosomes were also enriched with highly glycosylated PSMA analogous to LNCaP-derived exosomes. As controls, the exosomal markers (CD9 and TSG 101) were also highly enriched. Surprisingly, EpCAM was found to be at a low level in CWR22Rv1-derived exosomes, but detected at a higher level in CWR22Rv1 cells.

## Discussion

A plethora of empirical data support that tumor-derived exosomes can serve as cellular representatives or messengers carrying multiple forms of tumor-associated information including signaling molecules, tumor-markers and genetic factors, which may be an untapped potential source of cancer biomarkers for diagnostic or prognostic applications toward multiple cancer types (16). For prostate cancer, our study was carried out to explore whether prostate tumor-derived exosomes were enriched with PSMA, because PSMA has been widely studied and validated as an important biomarker for prostate cancer. Thus, two PSMA-positive prostate cancer cell lines: LNCaP (androgen-dependent) and CWR22Rv1 (androgen-independent) cells were employed in the present project. Although it has been reported that prostate tumor-derived exosomes can enrich biomarker PSMA (28,29), by using both of LNCaP and CWR22Rv1 cells, our data further confirmed the enrichment of exosomal PSMA without regard to androgen-dependence or -independence of PSMA-positive prostate cancer cells. To our surprise, our data revealed that exosomal PSMA is highly glyco sylated, and still retains about 24% enzymatic activity when compared to cellular PSMA. This evidence suggests that the origin of exosomal PSMA may be from internalization of mature (highly glycosylated) PSMA on the cell surface. The observed diminished activity of PSMA may be due to partial proteolysis or loss of native conformation under the low pH environment of endosomes; a result of the internalization process prior to fusing with multi-vesicular bodies (MVB) for exosome formation. Our data also suggest that there may be alternative fates for internalized PSMA: extracellular secretion through exosomes, recycling to the membrane surface or lysosomal digestion (4,6,30).

Currently, there are three major approaches for exosome isolation including ultracentrifugation, chemical precipitation and affinity-binding beads (31,32) which all have shortcomings. The first two approaches are void of specificity, and the last one is dependent on the binding-target protein. In example, EpCAM-based exosome-capture technology is not selective, suffering from contamination of normal tissue-derived exosomes, because EpCAM is widely expressed among a variety of human epithelial tissues, cancers, progenitor and stem cells (33). In contrast, highly-expressed PSMA is only found in prostate cancer cells (34). In fact, our group recently reported successful capture of PSMA-positive prostate cancer cells from blood samples using PSMA-based capture technology (35). Therefore, our data strongly support the development of a novel PSMA-based exosome capture technology platform for the accurate isolation of prostate tumor-derived exosomes from normal tissue-related exosomes.

In summary, our present data support the concept that prostate tumor-derived exosomes are highly enriched with tumor-marker biomolecules (especially membrane proteins, such as PSMA) representing characteristics of the original prostate cancer cells. Furthermore, characterization of tumor-derived exosomes may provide opportunities for the discovery of novel tumor-related biomarkers. We expect that developing a highly efficient, PSMA-based approach for tumor-derived exosome isolation will accelerate the innovation of non-invasive diagnostic or prognostic technologies for prostate cancer.
